# Integrating Climate Change Into Neurology Education: Preparing Future Physicians for a Warming World

**DOI:** 10.1212/NE9.0000000000200235

**Published:** 2025-07-29

**Authors:** Majd A. AbuAlrob, Adham Itbaisha

**Affiliations:** 1 Hamad General Hospital, Doha, Qatar; and; 2 Al-Quds University, Abu Dis, Palestine.

Neurologists must now contend with the profound neurologic consequences of climate change. Rising ambient temperatures, extreme weather events, and worsening air pollution have been linked to surges in stroke incidence, more severe cerebrovascular outcomes, and increased hospitalizations for migraine and dementia.^1^ Elevated ambient PM_2_._5_ levels measured 3 weeks earlier were associated with 21% higher odds of hospitalization for an MS relapse (OR 1.21; 95% CI 1.01–1.46), an effect that rose to 77% among patients younger than 30 years (OR 1.77; 95% CI 1.10–2.83).^2^ WHO models suggest that a 2–3°C rise in global temperatures could increase the population at risk of malaria by 3%–5%, potentially introducing cerebral malaria—the leading cause of acute febrile seizures and a major driver of epilepsy in sub-Saharan Africa—into regions previously naïve to the disease.^3^ Low-income and middle-income countries—despite contributing least to historic emissions—bear the steepest burden of climate-driven neurologic disease, thereby exacerbating existing health inequities.^2^

Although evidence increasingly links environmental shifts to neurologic health, planetary health topics remain scarcely taught: a 2019–20 global survey showed that fewer than 15% of medical schools had climate-health material in their required curriculum, only 6.8% of students felt that their training was adequate, and just 9.6% felt confident addressing climate-related health issues.^4^ This educational gap undermines our ability to prepare future neurologists to recognize, counsel, and manage climate-modulated illness—and to advocate for at-risk communities.

To address this shortfall, we propose 4 actionable strategies:

1. Embed climate case studies into ward rounds and didactics (e.g., heatstroke-induced intracerebral hemorrhage during heat waves and tick-borne encephalitis in expanding habitats).
2. Develop interdisciplinary modules in partnership with public health, focusing on air quality impacts on dementia and stroke and on vector-borne neuroinfections.
3. Offer elective rotations and scholarly projects on environmental neurology, mentored jointly by neurology and climate-health experts.
4. Define climate-neurology competencies within a competency-based medical education framework (e.g., patient counseling on heat avoidance and recognition of emerging pathogens) and integrate these into assessments and milestones.

By embedding these initiatives at both undergraduate and postgraduate levels, we can equip neurologists with the knowledge and skills to anticipate shifting disease patterns, mitigate emerging threats, and advocate for vulnerable populations. The neurology community has a professional and ethical obligation to incorporate climate science into our curricula. Failing to do so risks leaving the next generation of neurologists flat-footed in the face of preventable, climate-driven neurologic crises.

## References

[1] Sisodiya SM, Gulcebi MI, Fortunato F, et al. Climate change and disorders of the nervous system. Lancet Neurol. 2024;23(6):636-648. doi:10.1016/S1474-4422(24)00087-5 38760101

[2] Januel E, Dessimond B, Colette A, Annesi-Maesano I, Stankoff B. Fine particulate matter related to multiple sclerosis relapse in young patients. Front Neurol. 2021;12:651084. doi:10.3389/fneur.2021.651084 34093398 PMC8176031

[3] Gulcebi MI, Bartolini E, Lee O, et al. Climate change and epilepsy: insights from clinical and basic science studies. Epilepsy Behav. 2021;116:107791. doi:10.1016/j.yebeh.2021.107791 33578223 PMC9386889

[4] Navarrete-Welton A, Chen JJ, Byg B, et al. A grassroots approach for greener education: an example of a medical student-driven planetary health curriculum. Front Public Health. 2022;10:1013880. doi:10.3389/fpubh.2022.1013880 36225779 PMC9548693

